# Reflecting on earlier affected areas that shaped COVID-19 mental health efforts

**DOI:** 10.1007/s44192-022-00020-3

**Published:** 2022-07-11

**Authors:** Shawna K. Narayan, Vivian W. L. Tsang, Yue Qian

**Affiliations:** 1 Department of Psychiatry, Faculty of Medicine, University of British Columbia, #420-5950 University Boulevard, Vancouver, BC V6T 1Z3 Canada; 2 T.H. Chan School of Public Health, Harvard University, Boston, United States; 3 Department of Sociology, Faculty of Arts, University of British Columbia, Vancouver, Canada

## Abstract

The COVID-19 pandemic is a serious public health threat that many countries in the world are facing. While several measures are being taken to minimize the spread of infection, mental health efforts must address psychological challenges due to the pandemic. This commentary reflects on original research from earlier epicenters of COVID-19 and identifies effective practices and suggestions applicable to mental health interventions in the North American context. Tailored mental health services need to be provided for populations that are at high risk of infection. Suggested interventions targeting specific population groups, such as healthcare workers, COVID-19 patients, and vulnerable populations, are discussed.

## Introduction

The novel coronavirus disease (COVID-19) was declared a pandemic by the World Health Organization on March 11th, 2020, with 538,321,874 confirmed cases around the world and 6,320,599 confirmed deaths as of June 22nd, 2022 [1]. Global efforts to curb infection transmission include public health measures such as social distancing protocols [2]. In prior respiratory virus outbreaks, social isolation combined with public fear of falling ill resulted in known mental health consequences [3]. The outbreak of COVID-19 has caused public panic and mental health stress in China [4]. From worries of infection risk to social isolation and lack of financial security, the vast psychological impacts of COVID-19 have received increasing attention in cities where numerous acute cases have been resolved. In particular, challenges faced by health professionals, essential workers, and vulnerable populations extend past the physical consequences expected from the virus. Therefore, it is necessary to understand and analyze the effective mental health efforts for various populations. Learning from the experiences of earlier affected areas can help mitigate the psychological impacts of COVID-19 in the North American context and the case of future pandemics. This commentary reflects on the literature on mental health supports and recommendations from earlier areas affected by the COVID-19 pandemic. Therefore, the strategies and suggestions outlined in this editorial will be crucial to restructure ongoing mental health practices and inform new services both during and after COVID-19.

### Patients

COVID-19 patients, from those in self-isolation to those recovering from the disease, should be provided with mental healthcare that takes specific consideration of the isolation procedures and the changing severity of COVID-19 symptoms. Xiao and colleagues surveyed 170 individuals who self-isolated for 14 days in China [5]. Their findings suggested that low levels of social capital were associated with increased anxiety and stress levels [5]. Individuals in quarantine experienced boredom, loneliness, and anger. In addition, they may experience worsened anxiety and distress due to infection symptoms or adverse effects of treating somatic symptoms [6]. The study suggests that reliable information, expression of negative emotions, social connection, and maintaining a routine can better support patients in quarantine [6]. Most clinically stable COVID-19 patients also suffer significant post-traumatic stress symptoms (PTSS) prior to discharge [7]. Therefore, the lasting effect of PTSS must be addressed in psychological interventions for COVID-19 survivors through long-term follow-up assessments [7].

### Vulnerable populations

Yao, Chen, and Xu expressed concern that marginalized populations may be overlooked when considering the mental health consequences of the pandemic [8]. Research shows that the poor and vulnerable populations are often hit by disasters the most [9]. Qiu and colleagues surveyed 52,730 people affected by COVID-19 [10]. The survey outcomes provided recommendations for future interventions to focus on vulnerable groups such as migrant workers, youth, women, and the elderly [10]. In particular, individuals who are vulnerably housed, those with existing mental or physical illnesses, the elderly, and pregnant women may have difficulties accessing care either due to increased health risks of entering hospital or discrimination during the pandemic.

For individuals who are street entrenched with substance dependence and existing health conditions, the addition of COVID-19 creates not one but two public health emergencies – the overdose crisis alongside the pandemic [9]. First, as residents who are street entrenched face difficulties adhering to social distancing and basic hygiene protocols, they have a greater risk of infection [9, 11]. Residential instability and homelessness can also lead to more challenges to “identify, follow up, and treat those who are infected” [9]. Second, people who use drugs are more likely to have other comorbid health issues [9, 11]. The expansion of harm reduction supplies and safe drug supply is crucial during this time [12]. Third, quarantine protocols for people with existing diseases or mental health conditions mean that regular appointments or clinical follow-ups may be challenging to attend, thus causing more uncertainties for them compared to the general population [8]. Existing mental illness conditions may be exacerbated by fear, anxiety, and depression symptoms associated with the pandemic [8, 9, 17, 18].

Older adults are more likely to experience distress compared to others due to preexisting comorbidities and grief over the death of known loved ones [10, 13]. Furthermore, older adults may have limited access to online services and lack digital literacy to access mental health services [7, 14].

Heightened risk of infection and increased stress for pregnant women may result in side effects such as preeclampsia, depression, and preterm labour [15]. Additional concerns include changes to their birth plan, risk of infection to newborns, and challenges in receiving care [15]. Strategies to reduce anxiety include knowledge about transmission and symptoms of the virus, telehealth visits for pregnancy care, mental health check-ups, and offering women home birth services as an option [15].

### Healthcare workers

Healthcare workers are at risk of psychological distress due to long hours and high risk of infection, leading to stress, anxiety, burnout, and depressive symptoms [16]. In a cross-sectional study, healthcare workers in China reported depression, anxiety, insomnia, and distress symptoms while caring for patients with COVID-19 [17]. Zhang and colleagues found that insomnia symptoms were present in one-third of healthcare workers (N = 1563) [18]. In addition, Kang and colleagues summarized the enormous pressures healthcare workers face in Wuhan, China, including “high risk of infection and inadequate protection from contamination, overwork, frustration, discrimination, isolation, patients with negative emotions, a lack of contact with their families, and exhaustion” [16]. Similar findings were found in Gansu, China, with frontline workers dealing with high anxiety and depression symptoms [19]. Decision-making may also contribute to more significant mental distress for healthcare workers. In addition to ethical clinical dilemmas, healthcare workers also have to decide the equitable distribution of resources, as well as how to balance support and duty for family members and friends, the needs of patients, and personal wellness [18]. Several measures have been taken in Wuhan to minimize mental health issues, including a shift system for frontline workers to receive adequate rest, online platforms for medical advice, and psychological intervention teams to support staff’s mental wellbeing [16, 19]. Several of these methods have been adapted from the SARS outbreak, such as having multidisciplinary health teams, specialized psychiatric treatments, and effective communications [16].

## Conclusion

COVID-19 is putting tremendous stress on healthcare systems worldwide, including mental health. While social distancing interventions have been widely implemented to reduce new infection rates, potential unintended mental health outcomes, including increased suicide risk [20], must be prioritized as a national public health issue. While valuable efforts are being taken to mitigate some of the mental health challenges people face during the pandemic, the suggestions outlined from existing literature highlight the need to design *population-specific* mental health interventions and support effective COVID-19-related stress therapies for those impacted, such as peer-support groups and counselling sessions designed specifically for healthcare workers with moral injuries and mental health problems [19].

Key strategies identified by Wang and colleagues include (1) identifying high-risk groups based on sociodemographic characteristics, (2) understanding the immediate psychological needs of those affected by COVID-19, (3) providing accurate health information to reduce false news, (4) tailoring mental health interventions and services to suit the needs of the general population, and (5) adopting precautionary measures to prevent the spread of infection [21]. Understanding knowledge gaps, behavioural changes, and the impact of COVID-19 on mental health can support clinicians and policymakers in delivering more effective psychological interventions for the affected demographics. The dissemination of these best practices can contribute to the development of sustained, comprehensive, and equitable mental health care beyond COVID-19, and prepare for anticipated psychological impacts of future pandemics around the world.

## Data Availability

Not applicable.

## References

[1] World Health Organization. Coronavirus Disease 2019. 2021. https://www.who.int/emergencies/diseases/novel-coronavirus-2019/advice-for-public. Accessed June 23, 2022. 35767666

[2] World Health Organization. Mental health and psychosocial considerations during the COVID-19 outbreak. 2020. https://apps.who.int/iris/bitstream/handle/10665/331490/WHO-2019-nCoV-MentalHealth-2020.1-eng.pdf. Accessed April 21, 2020.

[3] Shah K, Kamrai D, Mekala H, Mann B, Desai K, Patel R (2020). Focus on mental health during the coronavirus (COVID-19) pandemic: applying learnings from the past outbreaks. Cureus.

[4] Bao Y, Sun Y, Meng S, Shi J, Lu L (2020). 2019-nCoV epidemic: address mental health care to empower society. Lancet.

[5] Xiao H, Zhang Y, Kong D, Li S, Yang N (2020). Social capital and sleep quality in individuals who self-isolated for 14 days during the coronavirus disease 2019 (COVID-19) outbreak in January 2020 in China. Med Sci Monit.

[6] Park S, Park YC (2020). Mental Health care measures in response to the 2019 novel coronavirus outbreak in Korea. Psychiatry Investig.

[7] Bo H, Li W, Yang Y, Wang Y, Zhang Q, Cheung T (2020). Posttraumatic stress symptoms and attitude toward crisis mental health services among clinically stable patients with COVID-19 in China. Psychol Med.

[8] Yao H, Chen J, Xu Y (2020). Patients with mental health disorders in the COVID-19 epidemic. Lancet Psychiatry.

[9] Druss BG (2020). Addressing the COVID-19 pandemic in populations with serious mental illness. JAMA Psychiat.

[10] Qiu J, Shen B, Zhao M, Wang Z, Xie B, Xu Y (2020). A nationwide survey of psychological distress among Chinese people in the COVID-19 epidemic: implications and policy recommendations. Gen Psychiatry.

[11] University of British Columbia. when crises collide: COVID-19 and overdose in the downtown eastside. 2020. https://www.arts.ubc.ca/news/when-crises-collide-covid-19-and-overdose-in-the-downtown-eastside/?fbclid=IwAR0JXYr6RPHtSVGYsh-oNPeySrYS6c66JSZpMEuZVCJ88SkYVusCcKQ5mvc. Accessed April 21, 2020.

[12] Karamouzian M, Johnson C, Kerr T (2020). Public health messaging and harm reduction in the time of COVID-19. Lancet Psychiatry.

[13] Yang Y, Li W, Zhang Q, Zhang L, Cheung T, Xiang Y (2020). Mental health services for older adults in China during the COVID-19 outbreak. Lancet Psychiatry.

[14] Tsai J, Wilson M (2020). COVID-19: a potential public health problem for homeless populations. Lancet Public Health.

[15] RashidiFakari F, Simbar M (2020). Coronavirus pandemic and worries during pregnancy; a letter to editor. Arch acad emerg med.

[16] Kang L, Li Y, Hu S, Chen M, Yang C, Yang BX (2020). The mental health of medical workers in Wuhan, China dealing with the 2019 novel coronavirus. Lancet Psychiatry.

[17] Lai J, Ma S, Wang Y, Cai Z, Hu J, Wei N (2020). Factors associated with mental health outcomes among health care workers exposed to coronavirus disease 2019. JAMA netw open.

[18] Zhang C, Yang L, Liu S, Ma S, Wang Y, Cai Z, Du H, Li R, Kang L, Su M (2020). Survey of insomnia and related social psychological factors among medical staff involved in the 2019 novel coronavirus disease outbreak. Front Psychiatry.

[19] Zhu J, Sun L, Zhang L, Wang H, Fan A, Yang B, Li W, Xiao S (2020). Prevalence and influencing factors of anxiety and depression symptoms in the first-line medical staff fighting against COVID-19 in gansu. Front Psychiatry.

[20] Chen Q, Liang M, Li Y, Guo J, Fei D, Wang L (2020). Mental health care for medical staff in China during the COVID-19 outbreak. Lancet Psychiatry.

[21] Wang C, Pan R, Wan X, Tan Y, Xu L, Ho CS, Ho RC (2020). Immediate psychological responses and associated factors during the initial stage of the 2019 coronavirus disease (COVID-19) epidemic among the general population in China. Int J Environ Res Public Health..

